# Effect of terminal chain length on the helical twisting power in achiral bent-core molecules doped in a cholesteric liquid crystal

**DOI:** 10.1039/c7ra11589j

**Published:** 2018-01-03

**Authors:** Byeong-Cheon Kim, Martin Walker, Seong-Yong Jo, Mark R. Wilson, Hideo Takezoe, Suk-Won Choi

**Affiliations:** Department of Advanced Materials Engineering for Information and Electronics (BK21Plus), Kyung Hee University Yongin-shi Gyeonggi-do 17104 Korea schoi@khu.ac.kr; Department of Chemistry, Durham University Lower Mountjoy, South Road DH13LE UK; Toyota Physical and Chemical Research Institute 41-1 Yokomichi Nagakute Aichi 480-1192 Japan

## Abstract

We prepared a homologous series of achiral bent-core (BC) liquid crystals with different terminal alkoxy chain lengths, *n* (BC-*n*), and evaluated the helical twisting power (HTP) of the BC-*n* doped in a cholesteric liquid crystal. The BC-*n* molecules with longer terminal chains showed larger HTPs. To interpret this striking phenomenon, a stochastic dynamics simulation was performed to determine the distribution of the chirality order parameters (*χ*) for BC molecules with *n* = 8–16. The distribution of *χ* for each simulated conformation varied with *n*, and the variation tendency was different for molecules with *n* < 12 and *n* > 12 despite the linear relationship between HTP and *n* in the experiment.

## Abstract 2

We prepared a homologous series of achiral bent-core (BC) liquid crystals with different terminal alkoxy chain lengths, *n* (BC-*n*), and evaluated the helical twisting power (HTP) of the BC-*n* doped in a cholesteric liquid crystal.

## Graphical abstract

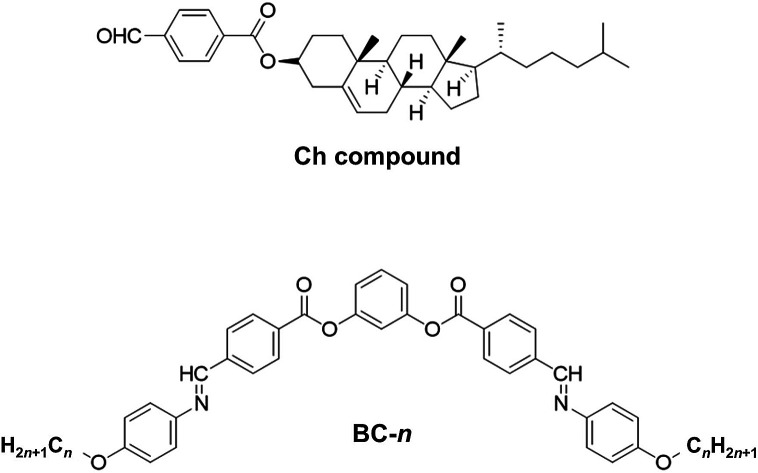

## Introduction

Since the discovery of polar switching in a liquid crystal (LC) phase composed of achiral bent-core (BC) molecules,^1^ a variety of intriguing phenomena related to BC molecules have attracted significant interest from many researchers.^2–4^ In this paper, we focus on a chirality-related phenomenon in BC molecular systems. Doping nematic LCs with chiral dopants induces a cholesteric (Ch) phase.^5^ In contrast, the doping of Ch LC with achiral molecules elongates the helical pitch of the Ch phase because the blending of achiral molecules dilutes the chirality of the system.^6^

Surprisingly, the helical pitch becomes shorter when the achiral BC molecules are blended into a Ch LC.^7^ This is quite unusual because the rational idea discussed above is no longer applicable. Paradoxically, achiral BC molecules sometimes behave as chiral ones. The unusual effect was qualitatively explained as follows: achiral BC molecules have two axially chiral conformers; when such molecules are dissolved in a chiral phase such as the Ch phase, one of the chiral conformers is predominantly selected and efficiently behaves as chiral ones, resulting in a decrease in the helical pitch. Many chiral conformations have been suggested based on Monte Carlo simulation.^8^ A similar phenomenon was also observed in a chiral smectic C phase (Sm*C and Sm*C_A_) doped with BC molecules^9^ and even with molecules with an ester linkage.^10,11^

As mentioned above, the helical pitch, *P*, of the Ch phase becomes shorter with increasing content of chiral molecules (dopant), *c*. The chirality-induction ability of chiral molecules is evaluated by helical twisting power (HTP), which is defined as1
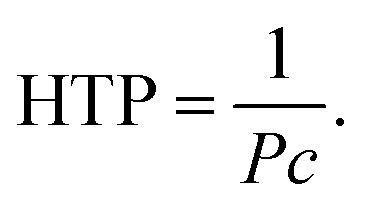


Recently, we reported that BC molecules with longer alkoxy chains tend to exhibit higher HTPs under chiral environments such as the Ch phase.^12^ In addition, preliminary calculations roughly supported the above-stated experimental results. In this study, we performed a systematic stochastic dynamics simulation to determine the distribution of chirality order parameters (*χ*) for BC homologues (BC-*n*) with different terminal chain lengths, *n* (*n* = 8–16). From the calculated distribution of *χ*, BC molecule's chiral conformations with extremely high HTPs can be identified. In the range from *n* = 8 to *n* = 12, the calculation result was consistent with our experimental result; the distribution became broader with *n*. However, the trend of distribution variation with *n* changes for *n* > 12. In this paper, we discuss the HTP behaviour depending on the terminal chain length based on the simulation.

## Experimental

**Fig. 1:**
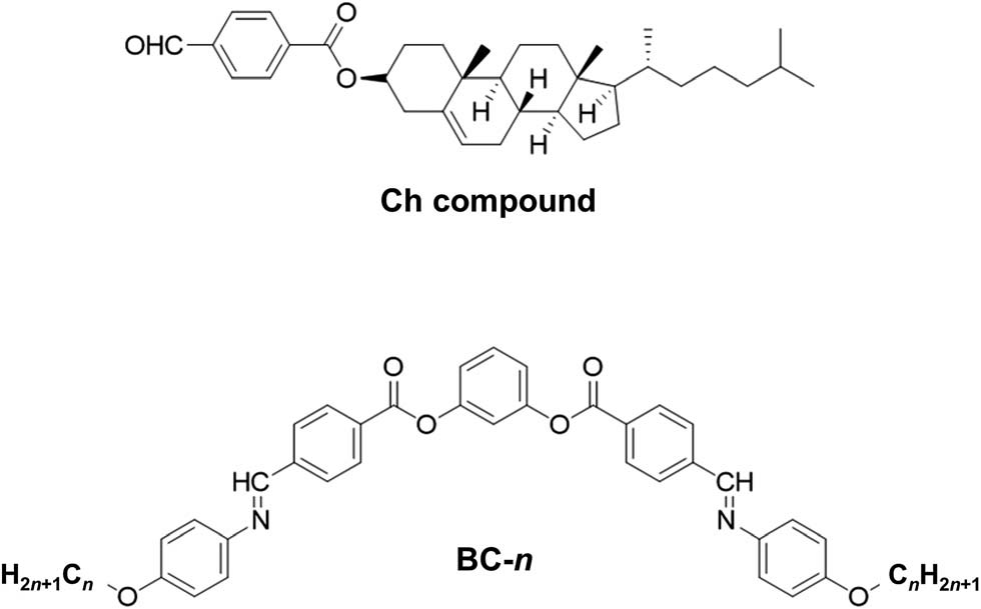
Chemical structures of the molecules used in this work.

For the doping experiments, we used a host Ch compound and six BC homologues (BC-*n*) with different terminal alkoxy chain lengths (*n* = 8, 9, 10, 12, 13, and 16). Fig. 1 shows the chemical structures of the molecules used in this study. The host Ch compound, which is the same compound employed in our previous work,^12^ possesses the Ch phase over a wide temperature range. We prepared mixture samples of the Ch compound (host) and the achiral BC-*n* (guest) in various weight fractions. The mixture samples were sandwiched between two glass plates, and the cells (sample thickness: about 10 μm) were cooled to solid state and then heated to the Ch phase using a temperature control unit (Mettler FP-82). The selective reflection spectra of the Ch phase, whose peak wavelength gives *P* multiplied by a refractive index, were measured using a multi-channel spectrometer (USB-2000, Ocean Optics). Here, we used a refractive index value of 1.5 irrespective of *n* and *c*, as in our previous work.^12^ The HTPs of BC homologues were evaluated using eqn (1).

### Simulation and analysis

Molecules with chain lengths *n* = 8 to *n* = 16 were initially equilibrated, using a modified General Amber Force Field (GAFF) potential,^13,14^ which has been optimised to reproduce the alkoxy chain conformations of LC molecules. The energy of each molecule was minimised with a steepest descent algorithm and considered optimised once the maximum force experienced by an atom was less than 10 kJ mol^−1^ nm^−1^. Once minimised, a stochastic dynamics simulation was performed at a temperature of 300 K with an inverse friction constant of 2 ps and time step of 1 fs. The acquisition of molecular configurations was started after a 50 ns equilibration period. The molecular configurations were collected every 10 ps for 5 μs, resulting in a total of 5 × 10^5^ configurations for analysis.

**Fig. 2:**
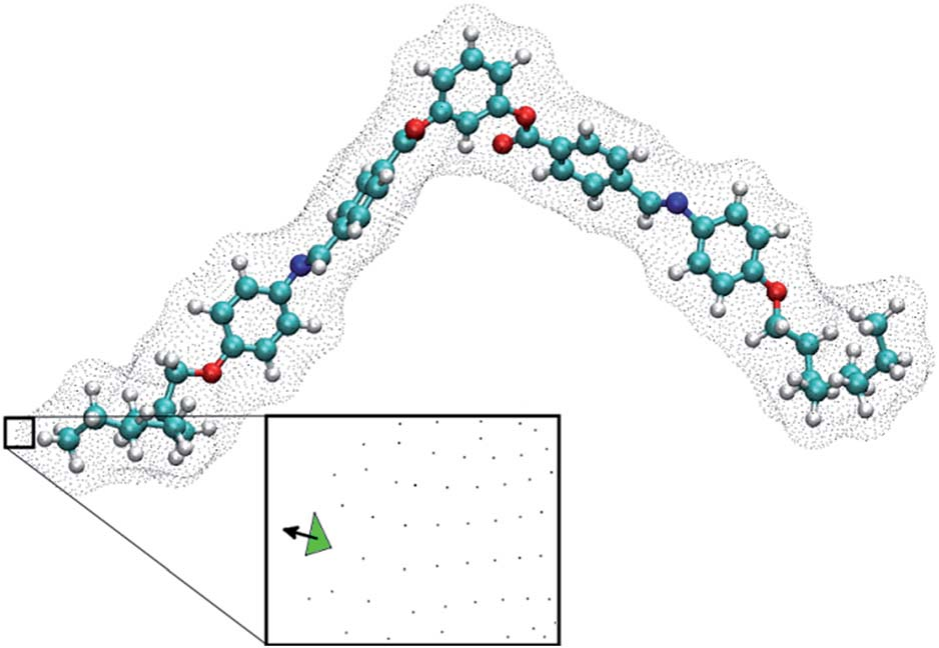
A BC-8 molecular configuration with its isosurface shown. The inset shows a plane generated from the isosurface (green triangle) and the vector normal to the isosurface. Integrating normal vectors over all surface points yields a surface tensor, **T**, (eqn (3), ref. 19), which gives the molecular principal axes (*T*_*xx*_, *T*_*yy*_, *T*_*zz*_) when diagonalised. Numerical integration over the molecular surface also yields the helicity tensor, **Q**, leading to the diagonal components (*Q*_*xx*_, *Q*_*yy*_, *Q*_*zz*_), in the principal axis system of **T** (eqn (4), ref. 19). Each molecular configuration results in a single chirality order parameter, *χ*, which is obtained from the helicity tensor and the ordering matrix (eqn (2)). Each molecular configuration results in a single chirality order parameter (*χ*).

Ferrarini *et al.*^15–18^ showed that molecular isosurfaces could be used to evaluate the chirality order parameter, *χ*, which is proportional to the helical twisting power of a molecule. Here,2
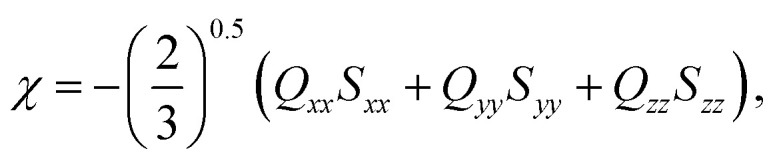
where *S*_*ii*_'s are the diagonal elements of the ordering matrix, **S**, and *Q*_*ii*_'s are the diagonal elements of the helicity tensor, **Q**, both are expressed in the principal axis system of the surface tensor **T** (see eqn (3), (4) and (6) in ref. 19). Diagonalisation of **T** yielded the components (*T*_*xx*_, *T*_*yy*_, *T*_*zz*_) that define the tendency of the molecular principal axes to align parallel (*T*_*zz*_) and perpendicular (*T*_*xx*_, *T*_*yy*_) to the rigid part of the BC mesogen. We used an isosurface corresponding to the van der Waals surface accessible to a spherical probe with a diameter of 5 Å. Isosurfaces were generated using the simple invariant molecular surface routine of Vorobjev and Hermans^20^ with a resolution of 10 dots per Å^2^. **S**, **Q**, **T** and *χ* are calculated for each molecular configuration. Fig. 2 shows an example of a molecular configuration (BC-8 here) with its isosurface.

## Results and discussion

**Fig. 3:**
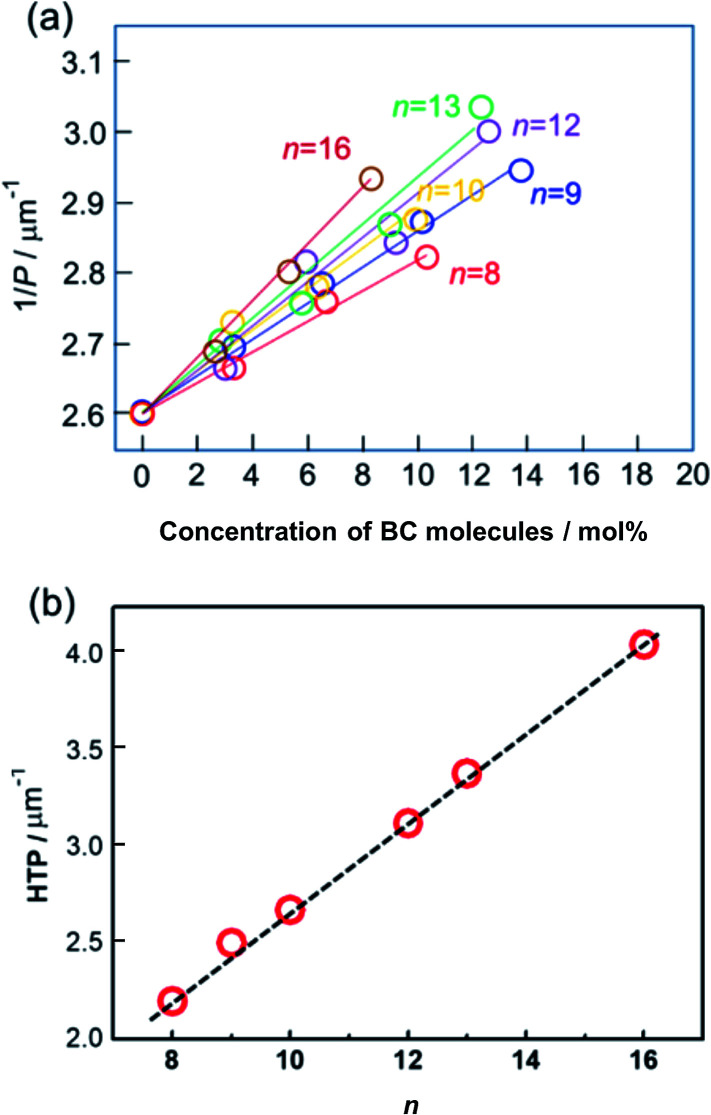
(a) Inverse of pitch (1/*P*) as a function of BC-*n* (*n* = 8, 9, 10, 12, 13, and 16) concentration (mol%). (b) HTP *vs. n*.

In this work, the phenomenon reported in our recent work was reproduced using the mixtures of Ch LC molecule and achiral BC-*n* LC with larger numbers of homologues (*n* = 8, 9, 10, 12, 13, and 16). Fig. 3(a) illustrates the inverse of pitch (1/*P*) at a reduced temperature, *T* − *T*_c_ = 20 °C, where *T*_c_ is the melting point of each mixture, as a function of BC-*n* content (mol%). The slope of each solid line exhibits the HTP of each doped BC molecule. The slope for each BC-*n* becomes steeper with increasing terminal-chain length. Thus, BC-*n* molecules with longer terminal chains show larger HTP, as described in Fig. 3(b). Interestingly, the evaluated HTPs for BC molecules exhibit a good linear relationship with terminal chain lengths. This indicates that the odd–even effect of HTP for the terminal chain length is negligible.

**Fig. 4:**
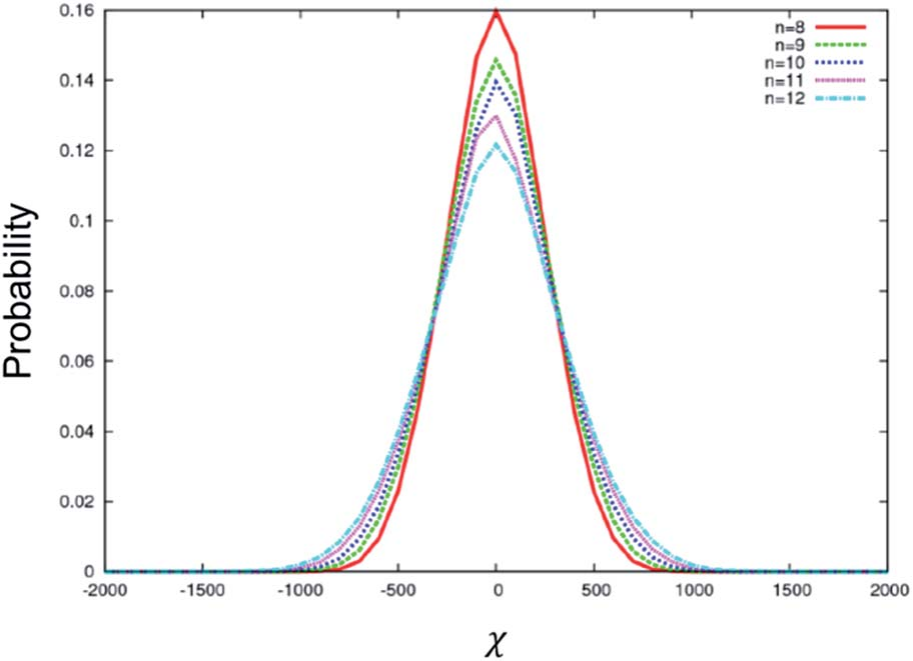
Distribution of *χ* for the conformations sampled by BC molecules with different terminal chain lengths from *n* = 8 to 12.

To understand the above experimental results, a stochastic dynamics simulation was carried out to determine the distribution of *χ* for the BC molecules used in this study. The range of values for *χ* implies that there are a variety of chiral conformations for each of these BC molecules, where a larger |*χ*| value induces a larger HTP value. A symmetrical distribution centred at *χ* = 0 represents an achiral molecule. Transient chiral configurations exist; however, there are equal numbers of left-handed configurations as right-handed configurations. The resulting molecule is therefore achiral. In a chiral medium, such as in the present case, the BC molecule will couple with the Ch medium, favouring a single handedness. Thus, a wider distribution of *χ* tends to exhibit larger HTP. Fig. 4 shows the distribution of *χ* for the conformations sampled by BC molecules with different terminal chain lengths from *n* = 8 to 12. The distribution is symmetric and centred at *χ* = 0. It is noted in Fig. 4 that the distribution of *χ* becomes wider, and the probability of an achiral molecule (*χ* = 0) decreases with increasing chain length. The maximum |*χ*| observed increases for larger *n*, while the number of achiral configurations decreases. Hence, if the chiral balance is slightly distorted under a chiral environment, a larger HTP can be obtained in BC-*n* with longer chain lengths for *n* = 8 to 12. These simulated results well explain the aforementioned experimental results. In addition, the odd–even effect of HTP for the terminal chain length is negligible, being consistent with the aforementioned experimental results.

**Fig. 5:**
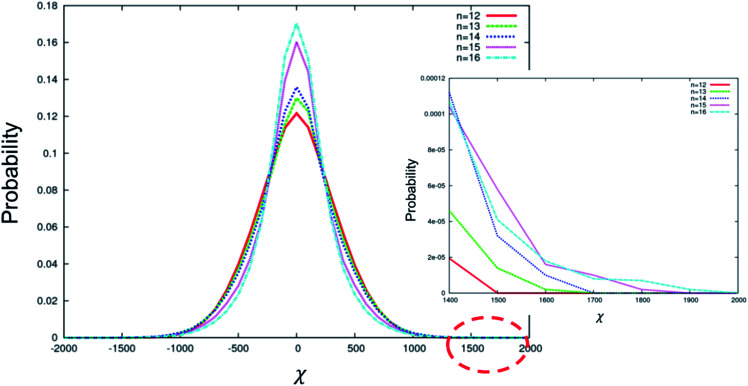
Distribution of *χ* for the conformations sampled by BC molecules with different terminal chain lengths from *n* = 12 to 16. A zoomed view of the range 1400 < *χ* < 2000 is shown in the inset.

Fig. 5 depicts the distribution of *χ* for the conformations sampled by BC molecules with longer terminal chain lengths from *n* = 12 to 16. In this range, the maximum |*χ*| increases with increasing *n* (inset in Fig. 5); however, the number of configurations around *χ* = 0 also increases with increasing *n*. We attribute this complex behaviour to the flexible alkyl arms, which can adopt many configurations, most of which are chiral. Longer flexible chains increase the maximum chirality that can be obtained from that chain. However, once the threshold length (*n* = 12) is reached, the chain can wrap around to interact with the rigid core. The core physically limits the space accessible to the arms, which in turn limits the possible configurations that can be accessed, altering the distribution of *χ*. Both the size of the core and the length of the flexible chains will explore this effect and subtly impact the distribution of configurations accessible to the molecule. We note that while the increase in maximum |*χ*| is in agreement with the experiment, the impact of the change on the shape of the *χ* distribution is not known. In addition, we cannot simply predict from our simulation that BC molecules with much longer terminal chains (*n* > 12) will always show higher HTP, as opposed to shorter chains (*n* ≤ 12), which behave in an uncomplicated manner. Our experimental results demonstrate that the HTP increases for the entire range of the arm lengths explored, from *n* = 8 to 16. This implies that the shape of the *χ* distribution has no impact on the HTP and that maximum |*χ*| is the only parameter correlated with the HTP.

## Conclusions

A series of achiral BC molecules with different terminal alkoxy chain lengths (*n* = 8–16) were prepared and doped into a host compound possessing the Ch phase. The evaluated HTPs for BC molecules exhibited good linear relationship with terminal chain lengths. In addition, the odd–even effect of the evaluated HTPs for the terminal chain length was negligible. To interpret this striking phenomenon, the distribution of the chirality order parameter, *χ*, for the conformations sampled by BC molecules with different terminal chain lengths was calculated. The behaviours of distribution changes for *n* = 8 to 12 and *n* = 12 to 16 are quite different despite the linear relationship between HTP and *n*. In the range from *n* = 8 to 12, the distribution becomes wider, strongly supporting our experimental result. However, the distribution changes exhibit a different trend in the range from *n* = 12 to 16 because of excessive bulkiness.

## Conflicts of interest

There are no conflicts to declare.

## Supplementary Material

## References

[cit1] Niori T., Sekine T., Watanabe J., Furukawa T., Takezoe H. (1996). J. Mater. Chem..

[cit2] Takezoe H., Takanishi Y. (2006). Jpn. J. Appl. Phys..

[cit3] Reddy R. A., Tschierske C. (2006). J. Mater. Chem..

[cit4] Takezoe H. and Eremin A., Bent-Shaped Liquid Crystals – Structures and Physical Properties, CRC Press, Boca Raton, 2017

[cit5] Kim K., Kim S., Jo S.-Y., Choi S.-W. (2015). J. Inf. Disp..

[cit6] Takezoe H. (2012). Top. Curr. Chem..

[cit7] Thisayukta J., Niwano H., Takezoe H., Watanabe J. (2002). J. Am. Chem. Soc..

[cit8] Earl D. J., Osipiv M. A., Takezoe H., Takanishi Y., Wilson M. R. (2005). Phys. Rev. E: Stat., Nonlinear, Soft Matter Phys..

[cit9] Gorecka E., Cepic M., Mieczkowski J., Nakata M., Takezoe H., Zeks B. (2003). Phys. Rev. E: Stat., Nonlinear, Soft Matter Phys..

[cit10] Choi S.-W., Fukuda K., Nakahara S., Kishikawa K., Takanishi Y., Ishikawa K., Watanabe J., Takezoe H. (2006). Chem. Lett..

[cit11] Jeong H. S., Tanaka S., Yoon D. K., Choi S.-W., Kim Y. H., Kawauchi S., Araoka F., Takezoe H., Jung H.-T. (2009). J. Am. Chem. Soc..

[cit12] Jo S.-Y., Kim B.-C., Jeon S.-W., Bae J.-H., Walker M., Wilson M., Choi S.-W., Takezoe H. (2017). RSC Adv..

[cit13] Boyd N. J., Wilson M. R. (2015). Phys. Chem. Chem. Phys..

[cit14] Boyd N. J., Wilson M. R. (2017). Phys. Chem. Chem. Phys..

[cit15] Ferrarini A., Moro G. J., Nordio P. L. (1995). Liq. Cryst..

[cit16] Ferrarini A., Moro G. J., Nordio P. L. (1996). Mol. Phys..

[cit17] di Matteo A., Todd S. M., Gottarelli G., Solladie G., Williams V. E., Lemieux R. P., Ferrarini A., Spada G. P. (2001). J. Am. Chem. Soc..

[cit18] Ferrarini A., Gottarelli G., Nordio P. L., Spada G. P. (1999). J. Chem. Soc., Perkin Trans. 2.

[cit19] Earl D. J., Wilson M. R. (2003). J. Chem. Phys..

[cit20] Vorobjev Y., Hermans J. (1997). Biophys. J..

